# Dissipation and Dietary Risk Assessment of the Fungicide Pyraclostrobin in Apples Using Ultra-High Performance Liquid Chromatography–Mass Spectrometry

**DOI:** 10.3390/molecules29184434

**Published:** 2024-09-18

**Authors:** Bin Wang, Lei Shi, Pengcheng Ren, Shu Qin, Jindong Li, Junli Cao

**Affiliations:** 1 Shanxi Center for Testing of Functional Agro-Products, Shanxi Agricultural University, Taiyuan 030031, China; 2 Shanxi Institute for Functional Food, Shanxi Agricultural University, Taiyuan 030031, China

**Keywords:** pyraclostrobin, UHPLC–MS/MS, dissipation, terminal residues, risk assessment

## Abstract

The fungicide pyraclostrobin is the main measure used to control apple alternaria blotch in production. To evaluate the potential dietary risks for consumers, the dissipation and terminal residues of pyraclostrobin were investigated using ultra-high performance liquid chromatography–mass spectrometry (UHPLC–MS/MS). Pyraclostrobin in apples was extracted by acetonitrile with 2% ammonia and then purified using primary secondary amine (PSA) and graphitized carbon black (GCB). The method showed good linearity within the concentration range of 0.005–0.1 mg L^−1^, with a coefficient of determination (R^2^) ≥ 0.9958. The recoveries ranged from 96.0% to 103.8%, with relative standard deviations (RSDs) between 0.8% and 2.3%. The limit of quantification (LOQ) was 0.01 mg kg^−1^. Pyraclostrobin dispersible oil suspension was applied in 12 apple fields across China according to good agricultural practices (GAPs). In Beijing and Shandong, the dissipation of pyraclostrobin followed first-order kinetic equations, with a half-life of 11 days. The terminal residues ranged from <0.01 to 0.09 mg kg^−1^. The national estimated daily intake (NEDI) of pyraclostrobin was compared with the acceptable daily intake (ADI), resulting in risk quotient (RQ_c_) of 80.8%. These results suggest that pyraclostrobin poses a low health risk to consumers under GAP conditions and according to recommended dosages.

## 1. Introduction

**Figure 1:**
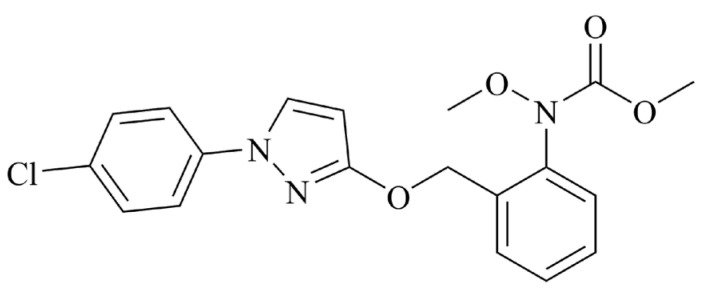
Structure of pyraclostrobin.

Apples are the most popular fruits among consumers, known for being rich in antioxidants, trace elements, vitamins, and other nutrients [1,2]. China is the world’s largest apple producer and consumer, with a planting area of 2,129,134 hectares in 2022, accounting for about half of the world’s apple production (https://www.fao.org/faostat/en/#compare (accessed on 29 July 2024)). However, apples are attacked by pests and diseases during their growth cycle, and pesticides need to be applied multiple times to protect farmers’ income. Pyraclostrobin (Figure 1), *N*-(2-phenyl)-*N*-methoxycarbamate, belongs to the broad-spectrum methacrylate fungicide used to control apple alternaria mali Roberts. It has antibacterial activity against almost all fungal plant pathogens by blocking the electron transfer between pathogen cytochromes, making it impossible for mitochondria to provide energy (ATP) to cells, ultimately leading to cell death [3].

There are 791 registered pyraclostrobin products in China, including 297 single-dose formulations (http://www.chinapesticide.org.cn/zwb/dataCenter (accessed on 29 July 2024)). However, excessive use of pesticides leads to pesticide residues, which seriously affect the quality of apple products and may increase potential risks to human health. Pyraclostrobin’s toxicity is widely recognized, as it initiates oxidative stress and apoptosis in zebrafish embryos [4,5], disrupts the antioxidant system in earthworms [6,7] (leading to irreversible toxicity), induces glutathione transferase activity and affects immobility in Daphnia magna [8], and has the potential to induce oxidative DNA damage and mitochondrial dysfunction in human hepatocytes [9]. The maximum residue limits (MRLs) of pyraclostrobin in apples set by the European Union, China, the United States, South Korea, and Japan are 0.5, 0.5, 1.5, 0.2, and 1 mg kg^−1^, respectively.

To avoid the potential harm of pyraclostrobin to public health, it is necessary to comprehensively study the residues of pyraclostrobin in apples and conduct a dietary risk assessment. As far as we know, there have been some reports of pyraclostrobin residues in fruits such as grapes [10,11], apricots [12], lemons [13], watermelons [14], bananas [15,16], strawberries [17,18], citrus [19], and apples [20]; information which is crucial for ensuring food safety. In these studies, LC–MS/MS and HPLC techniques were typically used to detect pyraclostrobin according to the different characteristics of the fruits. Fan et al. [21] studied pyraclostrobin’s dissipation and terminal residues in apple samples through field trials in the Beijing, Shandong, and Anhui provinces in China based on HPLC–MS/MS. With its increasing use, dietary risk assessments of pyraclostrobin residues in fruits are necessary to determine whether excessive use poses a hazard to consumers. However, the dietary risk assessment of pyrazole in apples under Good Agricultural Practice conditions has not been reported.

In this study, the residues of pyraclostrobin in 12 representative apples in China were determined, aiming to establish an accurate, simple, and sensitive UHPLC–MS/MS method for the determination of pyraclostrobin residues in apples; to assess the dietary intake risk of pyraclostrobin based on the final residues; to provide guidance for the dietary risk assessment of pyraclostrobin residues in apples; and to provide an effective analytical method for the determination of pyraclostrobin in apples.

## 2. Results and Discussion

### 2.1. Method Validation

**Figure 2:**
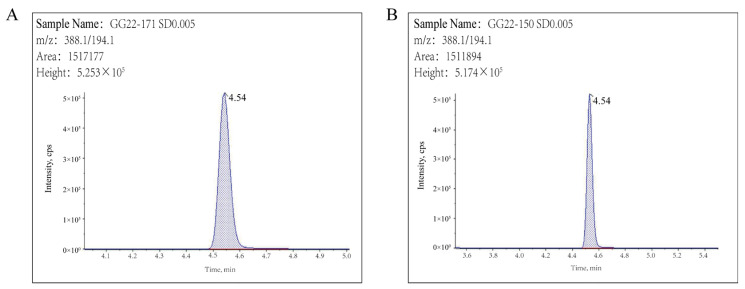
Chromatograms of 0.005 mg kg^−1^ pyraclostrobin in solvent (**A**) and blank apple (**B**).

QuEChERS pretreatment has become the preferred method for determining pesticide residues in food. In this study, a modified QuEChERS method was used to extract pyraclostrobin from apples, using 2% ammonia and acetonitrile as the extraction solvent, followed by sodium chloride (NaCl) salting out, PSA and GCB dispersive solid phase extraction cleanup, and finally ultra-high performance liquid chromatography–mass spectrometry (UHPLC–MS/MS) detection. As in our study, Li et al. used a modified QuEChERS method to extract pyraclostrobin, methyl thiophanate, carbendazim, and tebuconazole from apples [22]. As shown in Figure 2, pyraclostrobin was well separated, with a retention time of 4.54 min. The linearity, sensitivity, precision, accuracy, and matrix effect (ME) of the established method were verified according to the guidelines on pesticide residue trials (NY/T 788-2018) [23] and the European Union SANTE guidelines (SANTE/11312/2021) [24].

The accuracy and precision of the method were verified by five repeated recovery experiments at three spiked levels. As shown in Table 1, the recoveries of pyraclostrobin in apples were 96.0–103.8%, and the RSD was 0.8–2.3%, which conformed to the European Union SANTE guidelines (SANTE/11312/2021). The limit of quantification (LOQ) was defined as the minimum spiked level (0.01 mg kg^−1^). The recoveries of the method established by Fan et al. for detecting pyraclostrobin in apples ranged from 88.1% to 105.2%, with RSDs below 5.1% [21]. In contrast, the method we established was more accurate and stable, with recoveries ranging from 96.0 to 103.8% and RSDs below 2.3%.

ME refers to the interference of sample impurities with target compounds during ionization, thus affecting quantification accuracy [25]. ME may negatively impact the analysis of pyraclostrobin and must be evaluated during method validation. ME was usually evaluated by comparing the slopes of the matrix-matched standard curve to the slopes of the solvent standard curve. The pyraclostrobin standard solution was diluted with blank apple matrix (apples without pyraclostrobin treatment) and solvent to prepare 0.005, 0.01, 0.02, 0.05, and 0.1 mg L^−1^ standard solutions, which were measured by high-performance chromatography–tandem mass spectrometry. Blank apple samples and solvent were extracted and analyzed according to the established method, and no background interference was found at the retention time of pyraclostrobin. The matrix-matching calibration curve was y = 2.45253 × 10^8^x + 3.62017 × 10^5^, R^2^ = 0.9972 and the solvent calibration curve was y = 2.46766 × 10^8^x + 4.21324 × 10^5^, R^2^ = 0.9958, indicating an excellent linear relationship. The ME calculation result was −0.6%, indicating that the apple sample matrix had a weak inhibitory effect on pyraclostrobin. The ME was determined by the type of compound, extraction method and matrix type. There was considerable variability in matrix effects between different fruits. The ME of pyraclostrobin was 40% in strawberries [26] and 12.63% in pomegranates [27], using QuEChERS sample preparation combined with ultra-performance liquid chromatography–tandem mass spectrometry (UPLC–MS/MS). This study used the matrix-matching standard calibration method to eliminate the matrix effect.

In conclusion, the method established in this study was reliable, sensitive, and can be used to quantify pyraclostrobin in real apple samples.

### 2.2. Dissipation

**Figure 3:**
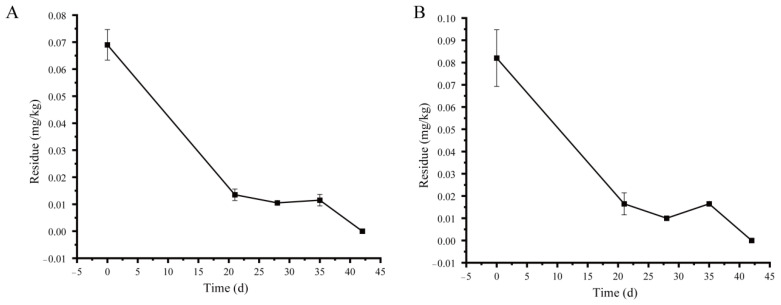
Dissipation curves of pyraclostrobin in apples in Beijing (**A**) and Shandong (**B**).

The validated method was applied to detect pyraclostrobin residues in field samples. 59 g/L pyraclostrobin dispersible oil suspension 800 times was sprayed three times, according to the maximum recommended dosage of 73.75 mg kg^−1^, with an interval of 7 days between applications. The initial depositions of pyraclostrobin in Shanxi, Beijing, Shandong, and Henan were 0.014, 0.069, 0.082, and 0.090 mg kg^−1^, respectively. The original deposition of pyraclostrobin on apples in Shanxi was significantly lower than in the other provinces. A variety of factors can affect the residual behavior of pesticides on crops, including the nature of the pesticide, fruit variety, climate, etc. The experimental apples in Shanxi were planted in 2013 and the trees are older than those in the other three places. The apples in Beijing and Shandong were planted in 2015, and those in Henan were planted in 2016. During the experiment, the average temperature (22.3 °C) and rainfall (202.4 mm) in Shanxi were higher than those in Beijing, where the average temperature was 21.2 °C and the average rainfall was 4.4 mm. Variances in the initial deposition of pyraclostrobin may be caused by soil type, apple variety, and temperature [28,29]. During the experiment, the average temperature in the four locations ranged from 21.2 °C to 27.9 °C. The apples in Shanxi, Beijing, Shandong, and Henan were mainly local varieties, namely Red Fuji, Red Fuji, Gala, and Red Star, respectively. There were significant differences in soil types among the Shanxi, Beijing, Shandong, and Henan provinces, with pH values of 8.48, 7.9, 6.9, and 7.9, cation exchange capacities of 13.92 cmol/kg, 13.8 cmol/kg, 9.3 cmol/kg, and 7.68 cmol/kg, and organic matter contents of 1.83%, 1.17%, 0.98%, and 1.29%, respectively. In previous studies, the half-life of pyraclostrobin was 12.8 days in banana pulp [15], 6.6–11.8 days in waxberries [30], 1.79–2.48 days in citrus [19], 17.8–25.9 days in grapes [10], and 3.27 days in watermelons [14]. Pyraclostrobin degraded rapidly in Shanxi and Anhui, and the residues were lower than LOQ at 28 days. Figure 3 shows the dissipation curves of pyraclostrobin in apples in Beijing and Shandong. The residues of pyraclostrobin in apples in Beijing and Shandong gradually decreased with time, consistent with the first-order kinetic equation. The dissipation equations were C_t_ = 0.069 × 10^−0.061t^ (R^2^ = 0.886) and C_t_ = 0.082 × 10^−0.061t^ (R^2^ = 0.7842); the half-life of pyraclostrobin was 11 days. Shandong and Beijing belong to a temperate semi-humid continental monsoon climate, while Shanxi and Henan belong to a temperate continental monsoon climate. Pyraclostrobin degrades rapidly in apples in Shanxi and Henan, and the half-life of pyraclostrobin in apples in Shandong and Beijing is 11 days, which may be related to climate. Consistent with our results, Magdalena et al. reported that the half-life of pyraclostrobin in apples was 11.5 days [31]. In another report, the original deposition of pyraclostrobin in apples was 0.07 to 0.53 mg kg^−1^, and the half-life was 4.3 to 8.3 days [32]. The existing research results showed that the half-life of pyraclostrobin in fruits was less than 30 days, which means that it is a degradable pesticide.

### 2.3. Final Residue

The samples were tested in batches according to their arrival time, and two quality control samples were added to each analysis. As shown in Table 2, the quality control results showed that the recoveries of pyraclostrobin in apples were 90.8–106.1%, indicating that the established detection method for pyraclostrobin was stable and accurate and the analysis results of the field trial samples were reliable.

Harvest intervals of 28 and 35 days were designed to monitor terminal residues of pyraclostrobin and recommend pre-harvest intervals (PHIs) for commercial formulations applied in apples. As shown in Table 3, at PHIs of 28 and 35 days, the terminal residues of pyraclostrobin in apples were <0.010–0.070 mg kg^−1^ and <0.010–0.048 mg kg^−1^, which were lower than the MRL (0.5 mg kg^−1^) recommended by China [33]. Therefore, according to the maximum recommended dosage of 73.75 mg kg^−1^ for spraying, 59 g L^−1^ pyraclostrobin dispersed oil suspension should be applied three times with an interval of 7 days between applications, and the recommended PHI is 28 days. These data can provide a reference for the rational use of pyraclostrobin in apples.

### 2.4. Dietary Risk Assessment

As a method, chronic risk quotient (RQ_c_) was the most commonly used parameter in dietary risk assessments of pesticide residues [34]. RQ_c_ was calculated by comparing the NEDI (national estimated daily intake) with the product of average body weight (bw) and ADI (acceptable daily intake). The weight of an Chinese adult was considered to be 63 kg [35]. An RQ_c_ greater than 100% indicates an unacceptable level of risk, whereas an RQc below 100% indicates an acceptable level of risk, and the larger the RQ_c_, the higher the risk [36]. The NEDI was calculated based on the typical Chinese dietary structure and the supervised trials median residue (STMR) or MRL of pyraclostrobin in registered crops. The ADI value of pyraclostrobin in GB2763-2021 was 0.03 mg kg^−1^ bw [33]. The MRL set by China was given priority. The MRL set by CAC and the United States was referenced if missing. The STMR of pyraclostrobin for 28 days was 0.012 mg kg^−1^. As shown in Table 4, the RQ_c_ of pyraclostrobin (80.8%) was less than 100%, indicating that pyraclostrobin will not pose a long-term risk to ordinary Chinese consumers when used in apples at the recommended dose and consumed according to the typical Chinese dietary structure.

## 3. Materials and Methods

### 3.1. Chemicals and Reagents

The certified reference standards of pyraclostrobin (purity 99.57%) were purchased from Dr. Ehrenstorfer (Augsburg, Germany). HPLC-grade acetonitrile was purchased from the Tedia Company, Inc. (Fairfield, CT, USA). LC–MS grade acetonitrile was provided by Thermo Fisher Scientific (Shanghai, China). Analytical grade ammonia was purchased from the Tianjin Damao Chemical Reagent Factory (Tianjin, China). Analytical grade anhydrous magnesium sulfate (MgSO_4_), sodium chloride (NaCl), disodium hydrogen citrate (C_6_H_6_Na_2_O_7_), sodium citrate (C_6_H_5_Na_3_O_7_), primary secondary amine (PSA, 40–60 μm), and graphitized carbon black (GCB, 40–60 μm) were purchased from Shimadzu Laboratory Equipment Co., Ltd. (Shanghai, China).

Weigh 10 ± 0.1 mg pyraclostrobin standard in a 10 mL volumetric flask and dissolve in mass spectrometry grade acetonitrile to prepare a standard stock solution (1000 mg L^−1^). Dilute the above standard stock solution with acetonitrile to prepare a 100 mg L^−1^ standard solution. Store at −18 °C until use. Dilute the above standard solution with acetonitrile and blank apple samples to prepare a series of standard solutions and matrix-matched standard solutions of 5, 10, 20, 50, and 100 μg L^−1^.

### 3.2. Chromatography Conditions

Pyraclostrobin was determined by ultra-high performance liquid chromatography–tandem triple quadrupole mass spectrometry (SCIEXAB TripleQuad 4500) (Foster City, CA, USA) in multiple reaction monitoring (MRM) mode and mass spectrometry analysis was performed in positive ion mode (ESI+). Pyraclostrobin was separated by ACQUITY UPLC^®^ HSST3 column (100 mm × 2.1 mm, 1.8 µm) (Waters, Milford, MA, USA), and the column temperature was 40 °C. The mobile phase was 4 mmol/L ammonium acetate aqueous solution (A) and methanol (B) containing 0.1% formic acid. The flow rate was 0.3 mL min^−1^ and the injection volume was 2 µL. The ion source temperature was 150 °C, and the cone voltage was 50 °C. Two daughter ions were selected as quantitative and qualitative ions: *m*/*z* 388.10 → 194.1 (18 eV) and 388.10 → 163.1 (36 eV).

### 3.3. Extraction and Purification Procedures

The QuEChERS method was developed by the United States Department of Agriculture in 2003 [37] and has been widely used in detecting pesticide residues in apples [38,39]. In this study, a modified QuEChERS method was used to extract pyraclostrobin residues in apples. 10.00 g apple samples were weighed into a 50 mL centrifuge tube. 20 mL acetonitrile containing 2% ammonia water was added to the centrifuge tube and ultrasonicated for 5 min. Then 4 g MgSO_4_, 1 g NaCl, 0.5 g disodium hydrogen citrate, and 1 g sodium citrate were added to each sample, oscillated for 1 min, and centrifuged at 3000 r min^−1^ for 3 min. 1.5 mL of supernatant was placed in a 2 mL centrifuge tube containing 142.5 mg MgSO_4_, 20 mg PSA, and 7.5 mg GCB, vortexed at 2500 r min^−1^ for 1 min, and centrifuged at 5000 r min^−1^ for 2 min. The supernatant was removed by syringe, passed through a 0.22 µm organic filter membrane, and stored in a sample injection vial for detection.

### 3.4. Method Validation

The method’s linearity, limit of quantification (LOQ), matrix effect, accuracy, and precision were validated according to the EU guidance document.

The pyraclostrobin standard solution was diluted to five concentrations (5, 10, 20, 50, 100 μg L^−1^) using blank apples and LC–MS grade acetonitrile to obtain matrix-matched and solvent-based calibration curves. Impurities in the sample matrix can interfere with the ionization of the target compound, thus affecting the accuracy of quantification. The matrix effect (ME%) was calculated as follows:

(1) $$ME\left(\%\right)=\frac{Smatrix-Ssolvent}{Ssolvent}\times 100\%$$

where S_matrix_ and S_solvent_ represent the slopes of the matrix-matched standard curve and the solvent standard curve, respectively.

The accuracy and precision of the method were evaluated by spike recovery experiment. The pyraclostrobin standard solution was spiked into blank apple samples at three concentrations (0.01, 0.1, and 0.5 mg kg^−1^) and five replicates were performed at each concentration level and allowed to stand for 1 h. The spiked samples were extracted and cleaned up according to the sample extraction and cleanup procedures are described in Section 3.3. The limit of quantification (LOQ) was defined as the lowest spike concentration.

### 3.5. Field Trials

Following the guidelines on pesticide residue trials (NY/T 788-2018) issued by the Ministry of Agriculture and Rural Affairs of China, the open field experiment was conducted in 12 different locations in China, including Shenyang, Liaoning (123.7 E, 42.7 N); Jinzhong, Shanxi (112.68 E, 37.55 N); Yuncheng, Shanxi (110.54 E, 35.06 N); Dingxi, Gansu (103.77 E, 35.64 N); Yinchuan, Ningxia (106.05 E, 38.48 N); Beijing (116.28 E, 40.22 N); Tai’an, Shandong (116.91 E, 36.00 N); Qingdao, Shandong (120.57 E, 36.41 N); Xinxiang, Henan (113.76 E, 34.98 N); Zhumadian, Henan (114.09 E, 33.07 N); Suzhou, Anhui (116.72 E, 344.30 N); and Kunming, Yunnan (102.37 E, 24.63 N). At least 4 apple trees should be in each experimental plot, with isolation zones between plots and a blank control set up. The average temperature and total precipitation information in 12 locations are shown in Table S1.

Terminal residue experiments were carried out in 12 locations. 59 g L^−1^ pyraclostrobin dispersible oil suspension 800 times was sprayed 3 times according to the recommended dosage of 73.75 mg kg^−1^, with intervals of 7 days. The control group was sprayed with water without pesticides. Mature apple samples were randomly collected from the experimental plots on the 28th and 35th days after the last spraying, with 2 independent samples collected each time. Detailed sample collection information is shown in Table S2.

Dissipation experiments were conducted in Jinzhong, Shanxi Province; Beijing and Tai’an, Shandong Province; and Xinxiang, Henan Province. 59 g L^−1^ pyraclostrobin dispersible oil suspension 800 times was sprayed three times, according to the maximum recommended dosage of 73.75 mg kg^−1^, with intervals of 7 days between applications. Apple samples were randomly collected from each plot at 0 (2 h), 21, 28, 35, and 42 days after the last spraying to evaluate the dissipation kinetics of pyraclostrobin. All samples were chopped, mixed, and divided into quarters, and two samples of no less than 200 g were taken, then labeled and stored in a −18°C freezer for further analysis. Detailed sample collection information is shown in Table S3.

### 3.6. Dissipation of Pyraclostrobin

The dissipation curve of pyraclostrobin was fitted by the first-order rate equation

C_t_ = C_0_ × exp(−kt) (2)

DT_50_ = In2/K (3)

where

C_t_ and C_0_ represent the residual concentration (mg kg^−1^) at time t (d) and the original deposition amount of pyraclostrobin;
k is the dissipation rate constant (d^−1^); and
DT_50_ indicates the degradation half-life of pyraclostrobin (d).

### 3.7. Chronic Dietary Risk Assessment

The chronic risk quotient (RQ_c_) was used to study the chronic dietary risk of pyraclostrobin, and the calculation formula was as follows:

(4) $$NEDI=\sum \left(STMR\times Fi\right)$$

(5) $${RQ}_{c}=\frac{NEDI}{ADI\times bw}$$

where

NEDI (mg kg^−1^) is the national estimated daily intake;
STMR (mg kg^−1^) is the supervised trials median residue of pyraclostrobin in apples;
Fi (kg) is the reference dietary intake;
ADI (mg kg^−1^ bw) is the acceptable daily intake; and
bw (63 kg) is the average body weight.

## 4. Conclusions

In this study, a sensitive and effective QuEChERS-UHPLC–MS/MS method was established to detect pyraclostrobin residues in apples. The established method showed satisfactory linearity, selectivity, trueness and precision validation parameters. The recoveries were 96.0–103.8% with RSD values below 2.3%. Field trials were conducted according to Good Agricultural Practice. Dissipation tests at four sites showed that the original deposition of pyraclostrobin in apples was between <0.010 and 0.093 mg kg^−1^, and the DT_50_ was 11 days in Beijing and Shandong. The results of field trials at 12 sites showed that the residues of pyraclostrobin in all samples were between <0.010 and 0.070 mg kg^−1^, which was lower than the MRL of 0.5 mg kg^−1^ recommended by China. Considering all registered crops for pyraclostrobin for dietary risk assessment, the calculated results showed that the threat to consumers at the recommended dose was negligible, with an RQ_c_ of 80.8%. Combined with the relevant MRL regulations and dietary intake risks, the PHI of 59 g L^−1^ pyraclostrobin dispersible oil suspension should be 28 days. This study can provide comprehensive risk assessment guidance for the rational use of pyraclostrobin in apple ecosystems.

## Figures and Tables

**Table 1:** Recoveries of pyraclostrobin in apples.

Spiked Level (mg kg^−1^)	Recoveries (%)						RSD (%)
	1	2	3	4	5	Average	
0.01	100.1	99.7	101.3	103.8	97.0	100.4	2.3
0.1	96.9	99.2	100.5	99.8	100.2	99.3	1.4
0.5	97.8	98.4	96.6	97.6	96.0	97.3	0.8

**Table 2:** Quality control (QC) of pyraclostrobin in real sample detection.

Spiked Level mg kg^−1^	QC Sample Analysis Date	Recoveries (%)		
		1	2	Average
0.10	31 August 2022	95.1	96.4	95.7
	21 September 2022	95.8	97.0	96.4
	13 October 2022	103.6	106.1	104.8
	24 October 2022	90.8	95.1	92.9
	3 November 2022	96.0	98.9	97.4

**Table 3:** The final residue of pyraclostrobin in apple samples.

Location	Terminal Residue (mg kg^−1^)				Supervised Trials Median Residue (STMR, mg kg^−1^)
	PHI = 28 days		PHI = 35 days		
	1	2	1	2	
Shenyang, Liaoning province	0.013	<0.010	<0.010	<0.010	0.012
Jinzhong, Shanxi Province	<0.010	<0.010	0.02	0.01	
Yuncheng, Shanxi Province	0.048	0.028	0.016	0.012	
Dingxi, Gansu province	0.013	0.012	<0.010	<0.010	
Yinchuan, Ningxia Province	0.031	0.042	0.022	0.016	
Changping, Beijing	0.011	<0.010	<0.010	0.013	
Tai’an, Shandong Province	0.01	<0.010	0.017	0.016	
Qingdao, Shandong Province	0.06	0.07	0.048	0.047	
Xinxiang, Henan Province	<0.010	<0.010	<0.010	<0.010	
Zhumadian, Henan province	<0.010	<0.010	<0.010	<0.010	
Suzhou, Anhui Province	0.019	0.014	0.013	<0.010	
Kunming, Yunnan Province	0.041	0.035	0.033	0.026	

**Table 4:** Chronic dietary risk assessment of pyraclostrobin.

Food Classification	Daily Consumption of a Particular Food (F_i_, kg)	Reference Residue Limits (mg kg^−1^)	National Estimated Daily Intake (NEDI, mg)	Acceptable Daily Intake×Average Body Weight (mg)	Chronic Risk Quotient (RQ_c_, %)
Rice and its products	0.2399	1 (China)	0.2399	ADI × 63	
Flour and its products	0.1385	0.2 (China)	0.0277		
Other cereals	0.0233	0.05 (China)	0.001165		
Tubers	0.0495	0.05 (China)	0.002475		
Dried beans (products)	0.016	0.2 (China)	0.0032		
Dark vegetables	0.0915	2 (China)	0.183		
light vegetables	0.1837	5 (China)	0.9185		
Pickles	0.0103				
Fruits	0.0457	0.012 (STMR, China)	0.0005484		
Nuts	0.0039				
Livestock and poultry	0.0795				
Milk and dairy products	0.0263				
Egg and its products	0.0236				
Egg and its products	0.0301				
Vegetable oil	0.0327	0.1 (China)	0.00327		
Animal oil	0.0087				
Sugar, starch	0.0044				
Salt	0.012	10 (China)	0.12		
Salt	0.009	3 (China)	0.027		
Total	1.0286		1.5268	1.8900	80.8%

## Data Availability

The data presented in this study are available from the authors upon request.

## Supplementary Materials

The following supporting information can be downloaded at https://www.mdpi.com/article/10.3390/molecules29184434/s1, Table S1: Average temperature and total precipitation information; Table S2: Apple sample collection for terminal residue experiment; Table S3: Apple sample collection for dissipation experiment.

## References

[1] Koutsos A., Tuohy K.M., Lovegrove J.A. (2015). Apples and cardiovascular health—Is the gut microbiota a core consideration?. Nutrients.

[2] Hyson D.A. (2011). A comprehensive review of apples and apple components and their relationship to human health. Adv. Nutr..

[3] Balba H. (2007). Review of strobilurin fungicide chemicals. J. Environ. Sci. Health Part B.

[4] Huang X., Yang S., Li B., Wang A., Li H., Li X., Luo J., Liu F., Mu W. (2021). Comparative toxicity of multiple exposure routes of pyraclostrobin in adult zebrafish (*Danio rerio*). Sci. Total Environ..

[5] Zhang C., Wang J., Zhang S., Zhu L., Du Z., Wang J. (2017). Acute and subchronic toxicity of pyraclostrobin in zebrafish (*Danio rerio*). Chemosphere.

[6] Hou K., Shi B., Liu Y., Lu C., Li D., Du Z., Li B., Zhu L. (2022). Toxicity evaluation of pyraclostrobin exposure in farmland soils and co-exposure with nZnO to *Eisenia fetida*. J. Hazard. Mater..

[7] Ma J., Cheng C., Du Z., Li B., Wang J., Wang J., Wang Z., Zhu L. (2019). Toxicological effects of pyraclostrobin on the antioxidant defense system and DNA damage in earthworms (*Eisenia fetida*). Ecol. Indic..

[8] Cui F., Chai T., Liu X., Wang C. (2017). Toxicity of three strobilurins (kresoxim-methyl, pyraclostrobin, and trifloxystrobin) on *Daphnia magna*. Environ. Toxicol. Chem..

[9] Wu M., Bian J., Han S., Zhang C., Xu W., Tao L., Li Z., Zhang Y. (2023). Characterization of hepatotoxic effects induced by pyraclostrobin in human HepG2 cells and zebrafish larvae. Chemosphere.

[10] Chen X., He S., Gao Y., Ma Y., Hu J., Liu X. (2019). Dissipation behavior, residue distribution and dietary risk assessment of field-incurred boscalid and pyraclostrobin in grape and grape field soil via MWCNTs-based QuEChERS using an RRLC-QqQ-MS/MS technique. Food Chem..

[11] Zhao P., Liu R., Yuan L. (2024). Dissipation, Residue and Human Dietary Risk Assessment of Pyraclostrobin and Cyazofamid in Grapes Using an HPLC-UV Detector. Foods.

[12] Dost K., Oksuz M., Cittan M., Mutlu B., Tural B. (2023). Determination of boscalid, pyraclostrobin and trifloxystrobin in dried grape and apricot by HPLC/UV method. J. Food Compos. Anal..

[13] Dominguez A.N., Jimenez L.E., Alvarez R.M.S. (2023). Rapid detection of pyraclostrobin fungicide residues in lemon with surface-enhanced Raman spectroscopy. J. Food Meas. Charact..

[14] Lv L., Su Y., Dong B., Lu W., Hu J., Liu X. (2022). Dissipation Residue Behaviors and Dietary Risk Assessment of Boscalid and Pyraclostrobin in Watermelon by HPLC-MS/MS. Molecules.

[15] Lin S., Yang L., Zheng Q., Wang Y., Cheng D., Zhang Z. (2022). Dissipation and distribution of pyraclostrobin in bananas at different temperature and a risk assessment of dietary intake. Int. J. Environ. Anal. Chem..

[16] Yang M., Zhang J., Zhang J., Rashid M., Zhong G., Liu J. (2018). The control effect of fungicide pyraclostrobin against freckle disease of banana and its residue dynamics under field conditions. J. Environ. Sci. Health Part B.

[17] Malhat F., Saber E.-S., Shokr S.A.E., Ahmed M.T., Amin A.E.-S. (2019). Consumer safety evaluation of pyraclostrobin residues in strawberry using liquid chromatography tandem mass spectrometry (LC-MS/MS): An Egyptian profile. Regul. Toxicol. Pharmacol..

[18] Valera-Tarifa N., Santiago-Valverde R., Hernández-Torres E., Martínez-Vidal J., Garrido-Frenich A. (2020). Development and full validation of a multiresidue method for the analysis of a wide range of pesticides in processed fruit by UHPLC-MS/MS. Food Chem..

[19] Liu X., Yang Y., Chen Y., Zhang Q., Lu P., Hu D. (2019). Dissipation, residues and risk assessment of oxine-copper and pyraclostrobin in citrus. Food Addit. Contam. Part A Chem. Anal. Control Expo. Risk Assess..

[20] Zarębska M., Hordyjewicz-Baran Z., Wasilewski T., Zajszły-Turko E., Stanek N. (2022). A new LC-MS method for evaluating the efficacy of pesticide residue removal from fruit surfaces by washing agents. Processes.

[21] Fan X., Zhao S., Chen X., Hu J. (2018). Simultaneous Determination of Pyraclostrobin, Prochloraz, and its Metabolite in Apple and Soil Via RRLC-MS/MS. Food Anal. Methods.

[22] Li P., Sun P., Dong X., Li B. (2020). Residue analysis and kinetics modeling of thiophanate-methyl, carbendazim, tebuconazole and pyraclostrobin in apple tree bark using QuEChERS/HPLC–VWD. Biomed. Chromatogr..

[23] Ministry of Agriculture and Rural Affairs of the People’s Republic of China (2018). Guideline for the Testing of Pesticide Residues in Crops.

[24] European Commission (2021). Guidance Document on Analytical Quality Control and Method Validation Procedures for Pesticides Residues and Analysis in Food and Feed.

[25] Matuszewski B.K., Constanzer M., Chavez-Eng C. (2003). Strategies for the assessment of matrix effect in quantitative bioanalytical methods based on HPLC−MS/MS. Anal. Chem..

[26] Wang H., Ping H., Liu Q., Han P., Guo X. (2022). Determination of Pesticide Residues in Strawberries by Ultra-performance Liquid Chromatography-Tandem Mass Spectrometry. Food Anal. Methods.

[27] Naik R.H., Pallavi M.S., Nandini, Shwetha A., Bheemanna M., Nidoni R.U. (2022). Simultaneous determination of pesticide residues in pomegranate whole fruit and arils using LC-MS/MS. Food Chem..

[28] Fan X., Zhao S., Hu J. (2019). Dissipation behavior and dietary risk assessment of lambda-cyhalothrin, thiamethoxam and its metabolite clothianidin in apple after open field application. Regul. Toxicol. Pharmacol..

[29] Tian F., Lu J., Qiao C., Wang C., Pang T., Guo L., Li J., Pang R., Xie H. (2024). Dissipation behavior and risk assessment of imidacloprid and its metabolites in apple from field to products. Chemosphere.

[30] Jianzhong Y., Liezhong C., Jiayin H., Ruixian Y., Xiuqing H., Xueping Z. (2020). Residue and dissipation dynamics of pyraclostrobin in waxberry (*Myrica rubra*) and soil. Chin. J. Pestic. Sci..

[31] Podbielska M., Szpyrka E., Piechowicz B., Sadło S., Sudoł M. (2018). Assessment of boscalid and pyraclostrobin disappearance and behavior following application of effective microorganisms on apples. J. Environ. Sci. Health Part B.

[32] Yan Q., Pengfei D., Yue Z., Shanshan W., Maojun J., Jing W. (2017). Determination and dissipation dynamics of cuppric nonyl phenolsulfonate and pyraclostrobin in apples and soil. Chin. J. Pestic. Sci..

[33] (2021). National Food Safety Standard-Maximum Residue Limits for Pesticides in Food.

[34] Dong M., Ma L., Zhan X., Chen J., Huang L., Wang W., Zhao L. (2019). Dissipation rates and residue levels of diflubenzuron and difenoconazole on peaches and dietary risk assessment. Regul. Toxicol. Pharmacol..

[35] Li R., Men X., Li R., Liu T., Liang H., Fang F., Sun-Waterhouse D., Wang Y. (2023). Residue behaviors and dietary risk of cyazofamid in turnip, onion and romaine lettuce assessed by a QuEChERS-LC-MS/MS method. Food Sci. Hum. Wellness.

[36] Dong X., Tong Z., Chu Y., Sun M., Wang M., Gao T., Duan J. (2019). Dissipation of prothioconazole and its metabolite prothioconazole-desthio in rice fields and risk assessment of its dietary intake. J. Agric. Food Chem..

[37] Anastassiades M., Lehotay S.J., Štajnbaher D., Schenck F.J. (2003). Fast and easy multiresidue method employing acetonitrile extraction/partitioning and “dispersive solid-phase extraction” for the determination of pesticide residues in produce. J. AOAC Int..

[38] Lehotay S.J., Son K.A., Kwon H., Koesukwiwat U., Fu W., Mastovska K., Hoh E., Leepipatpiboon N. (2010). Comparison of QuEChERS sample preparation methods for the analysis of pesticide residues in fruits and vegetables. J. Chromatogr. A.

[39] Machado I., Gérez N., Pistón M., Heinzen H., Cesio M.V. (2017). Determination of pesticide residues in globe artichoke leaves and fruits by GC–MS and LC–MS/MS using the same QuEChERS procedure. Food Chem..

