# Supplementary material for: Dissipation and Dietary Risk Assessment of the Fungicide Pyraclostrobin in Apples Using Ultra-High Performance Liquid Chromatography–Mass Spectrometry
Source: Molecules. 2024 Sep 18;29(18):4434. doi: 10.3390/molecules29184434 (PMC11434584; doi:10.3390/molecules29184434)
Supplement: Supplementary file 1 [file molecules-29-04434-s001.zip › molecules-3184318-supplementary.pdf]

Table S1. Average temperature and total precipitation information

Location	Average temperature (℃)	Total precipitation （mm）
Shenyang, Liaoning province	14.8	28.07
Jinzhong, Shanxi Province	22.3	202.4
Yuncheng, Shanxi Province	27.2	108.92
Dingxi, Gansu province	17.4	89
Yinchuan, Ningxia Province	24.5	13.3
Changping, Beijing	21.2	4.4
Tai'an, Shandong Province	27.9	1574.8
Qingdao, Shandong Province	22.5	1582.2
Xinxiang, Henan Province	27.7	/
Zhumadian, Henan province	27.8	/
Suzhou, Anhui Province	20.3	15.5
Kunming, Yunnan Province	21.4	134

Table S2 Apple sample collection for terminal residue experiment

Location	Treat	Samples Number	
		PHI=28 days	PHI=35 days
Shenyang, Liaoning province	Control	CK-1A,CK-2A，CK-1B,CK-2B	CK-3A,CK-4A，CK-3B,CK-4B
	Spraying pyraclostrobin	1A,2A，1B,2B	3A,4A，3B,4B
Jinzhong, Shanxi Province	Control	CK-1A,CK-2A，CK-1B,CK-2B	CK-3A,CK-4A，CK-3B,CK-4B
	Spraying pyraclostrobin	1A,2A，1B,2B	3A,4A，3B,4B
Yuncheng, Shanxi Province	Control	CK-1A,CK-2A，CK-1B,CK-2B	CK-3A,CK-4A，CK-3B,CK-4B
	Spraying pyraclostrobin	1A,2A，1B,2B	3A,4A，3B,4B
Dingxi, Gansu province	Control	CK-1A,CK-2A，CK-1B,CK-2B	CK-3A,CK-4A，CK-3B,CK-4B
	Spraying pyraclostrobin	1A,2A，1B,2B	3A,4A，3B,4B
Yinchuan, Ningxia Province	Control	CK-1A,CK-2A，CK-1B,CK-2B	CK-3A,CK-4A，CK-3B,CK-4B
	Spraying pyraclostrobin	1A,2A，1B,2B	3A,4A，3B,4B
Changping, Beijing	Control	CK-1A,CK-2A，CK-1B,CK-2B	CK-3A,CK-4A，CK-3B,CK-4B
	Spraying pyraclostrobin	1A,2A，1B,2B	3A,4A，3B,4B
Tai'an, Shandong Province	Control	CK-1A,CK-2A，CK-1B,CK-2B	CK-3A,CK-4A，CK-3B,CK-4B
	Spraying pyraclostrobin	1A,2A，1B,2B	3A,4A，3B,4B
Qingdao, Shandong Province	Control	CK-1A,CK-2A，CK-1B,CK-2B	CK-3A,CK-4A，CK-3B,CK-4B
	Spraying pyraclostrobin	1A,2A，1B,2B	3A,4A，3B,4B
Xinxiang, Henan Province	Control	CK-1A,CK-2A，CK-1B,CK-2B	CK-3A,CK-4A，CK-3B,CK-4B
	Spraying pyraclostrobin	1A,2A，1B,2B	3A,4A，3B,4B
Zhumadian, Henan province	Control	CK-1A,CK-2A，CK-1B,CK-2B	CK-3A,CK-4A，CK-3B,CK-4B
	Spraying pyraclostrobin	1A,2A，1B,2B	3A,4A，3B,4B
Suzhou, Anhui Province	Control	CK-1A,CK-2A，CK-1B,CK-2B	CK-3A,CK-4A，CK-3B,CK-4B
	Spraying pyraclostrobin	1A,2A，1B,2B	3A,4A，3B,4B
Kunming, Yunnan Province	Control	CK-1A,CK-2A，CK-1B,CK-2B	CK-3A,CK-4A，CK-3B,CK-4B
	Spraying pyraclostrobin	1A,2A，1B,2B	3A,4A，3B,4B

Table S3 Apple sample collection for dissipation experiment

**Location**	**Sampling time (sample number)**
Jinzhong, Shanxi Province	2 hours(2), 21d(2), 28d(2),35d(2) and 42d(2)
Changping, Beijing	2 hours(2), 21d(2), 28d(2),35d(2) and 42d(2)
Tai'an, Shandong Province	2 hours(2), 21d(2), 28d(2),35d(2) and 42d(2)
Xinxiang, Henan Province	2 hours(2), 21d(2), 28d(2),35d(2) and 42d(2)
